# Synergistic Function between Phosphorus-Containing Flame Retardant and Multi-Walled Carbon Nanotubes towards Fire Safe Polystyrene Composites with Enhanced Electromagnetic Interference Shielding

**DOI:** 10.3390/ijms232113434

**Published:** 2022-11-03

**Authors:** Ruizhe Huang, Caiqin Gao, Yongqian Shi, Libi Fu, Yuezhan Feng, Wei Shui

**Affiliations:** 1 College of Environment and Safety Engineering, Fuzhou University, 2 Xueyuan Road, Fuzhou 350116, China; 2 College of Civil Engineering, Fuzhou University, 2 Xueyuan Road, Fuzhou 350116, China; 3 Key Laboratory of Materials Processing and Mold Ministry of Education, National Engineering Research Center for Advanced Polymer Processing Technology, Zhengzhou University, Zhengzhou 450002, China

**Keywords:** flame retardancy, electromagnetic interference shielding, multiple effects, multi-walled carbon nanotube

## Abstract

As a universal polymer material, polystyrene (PS) is widely applied in electrical devices and construction. Thus, it is necessary to improve the flame retardancy and electromagnetic shielding properties of PS material. In this work, PS/silicon-wrapped ammonium polyphosphate/Inorganic acid-treated multi-walled carbon nanotubes composites (PS/SiAPP/aMWCNT, abbreviated as PAC) were prepared via methods of filtration-induced assembly and hot-pressing. Morphology and structure characterization demonstrated that SiAPP and aMWCNT had good dispersion in PS and excellent compatibility with the PS matrix. Thermogravimetric analysis revealed that the addition of aMWCNT to PS improved its thermal stability and carbon-forming characteristics. The peak heat release rate, the peak carbon monoxide production rate, and the peak smoke production rate of the PAC10 composite decreased by 53.7%, 41.9%, and 45.5%, respectively, while its electromagnetic shielding effectiveness reached 12 dB. These enhancements were attributed to the reason that SiAPP and aMWCNT synergistically catalyzed the char generation and SiAPP produced free radical scavengers and numbers of incombustible gases, which could decrease the oxygen concentration and retard the combustion reaction. Therefore, the assembled PS/SiAPP/aMWCNT system provides a new pathway to improve the flame retardant and electromagnetic shielding properties of PS.

## 1. Introduction

Polystyrene (PS) is a multi-purpose thermoplastic polymer material with low density, remarkable formability, mechanical durability, and thermal resistance [1]. Thus, PS is extensively used in electronic equipment, automotive, construction, and aerospace fields [2]. However, PS is a kind of highly flammable material, and produces a great deal of smoke, droplets, and toxic gases (such as carbon monoxide, carbon dioxide, etc.) when burned [3,4]. The smoke and toxic gases generated by combustion are harmful to human health, which restricts the further application of PS. Thus, it is necessary to improve the flame-retardant performance of PS to reduce the occurrence of fire accidents. Moreover, with the rapid development of electronic technology, mobile phones, computers, wearable electronic devices, and recently launched 5G devices are widely used in public daily life. These electronic devices will produce magnanimous electromagnetic radiation, which will interfere with the normal operation of other electronic devices and even damage the human body [5,6]. Polymer materials can be used as the shell of electronic equipment to improve the electromagnetic shielding performance and effectively prevent the interference of external electromagnetic waves. PS as an insulator will be completely penetrated by electromagnetic waves, which limits its electromagnetic shielding performance [7]. Meanwhile, electromagnetic shielding materials need to reduce fire hazards in practical applications. Therefore, it is important to construct PS composites with excellent flame-retardant and electromagnetic shielding properties to extend the application prospects of PS.

Recently, the addition of phosphorus-containing flame retardants has been shown to be an effective strategy to improve the fire resistance of polymer materials, which can produce a carbonized layer with a more stable structure when the polymer is heated [8,9]. Ammonium polyphosphate (APP) has received a great deal of attention in the application field of flame-retardant materials due to its low toxicity and low smoke production, which can significantly enhance the flame-retardant properties of polymers and reduce the release of toxic gases [10,11,12,13]. The effects of APP on the flame-retardant properties of PS have been well studied. Wang et al. prepared PS/poly(1,3,5-triazine-2-aminoethanol diethylenetriamine) and APP [9]. The results showed that the flame-retardant and smoke-inhibition properties of PS composites were prominently better than those of pure PS. In addition, Shi et al. investigated the synergistic effect between APP and graphitic carbon nitride (g-C_3_N_4_) for pure PS and found that combining APP with g-C_3_N_4_ or its derivatives could significantly improve the flame-retardant properties of PS [14,15,16,17,18]. These results showed that the fire retardancy of PS could be improved by combining APP with other flame retardants. However, studies have found that APP has poor compatibility with polymeric materials, and individually adding APP to improve the flame-retardant properties of PS is not an effective method, which limits the wide application of APP [19]. Currently, improving the water resistance and interfacial adhesion of APP through surface modification or combination with other flame-retardant additives receives extensive attention. Among them, a surfactant, a coupling agent, and microencapsulation were applied to functionalize APP [20,21,22,23,24,25,26,27,28,29,30,31,32,33]. The modification of APP using coupling agents not only improves the hydrophobicity of APP but also enhances its compatibility with the matrix.

Multi-wall carbon nanotubes (MWCNTs), as a typical one-dimensional, high-conductivity, nanostructured material, is an ideal conductive filler for preparing high-performance electromagnetic interference shielding composites due to its advantages of a small diameter, a high spread ratio, electrical conductivity, and high mechanical strength [34,35]. Yang et al. prepared PS/MWCNT composites, and the electromagnetic interference (EMI) shielding properties of PS composites with a loading of 7 wt.% MWCNT reached 20 dB [36]. Anju Gupta et al. investigated the EMI shielding effectiveness (SE) of poly (trimethyl terephthalate)/MWCNT composites and found that the EMI shielding effectiveness (SE) of the composite reached 42 dB in the Ku band with the addition of 10 wt.% MWCNT [37]. Abilash Mende Anjaneyalu et al. fabricated PS composites with a separated structure by adding MWCNT and nickel nanowires (NINW) to the PS matrix [35]. The EMI SE of PS/2.0 vol.% MWCNT nanocomposites with a thickness of 1.1 mm at X band was approximately 16.6 dB, whereas the EMI SE of PS/2.0 vol.% MWCNT/0.5 vol.% NINW nanocomposites at the band was 23.2 dB. Obviously, the electromagnetic shielding performance of PS was further improved after adding NINW. These studies indicated that the addition of MWCNT can indeed enhance the electromagnetic shielding performance of polymers.

In this work, the MWCNT and silicon-wrapped APP (SiAPP) were introduced into the PS matrix to construct PS composites (PS/SiAPP/aMWCNT) by filtration-induced assembly and hot-pressing methods. The morphology, chemical structure, thermal stability, flame retardancy, smoke suppression, and electromagnetic interference shielding properties of PS/SiAPP/aMWCNT composites were investigated. The mechanisms for enhancing flame-retardant and electromagnetic interference shielding performances of PS composites were explored.

## 2. Results

### 2.1. Morphology and Structure Characterization

**Figure 1:**
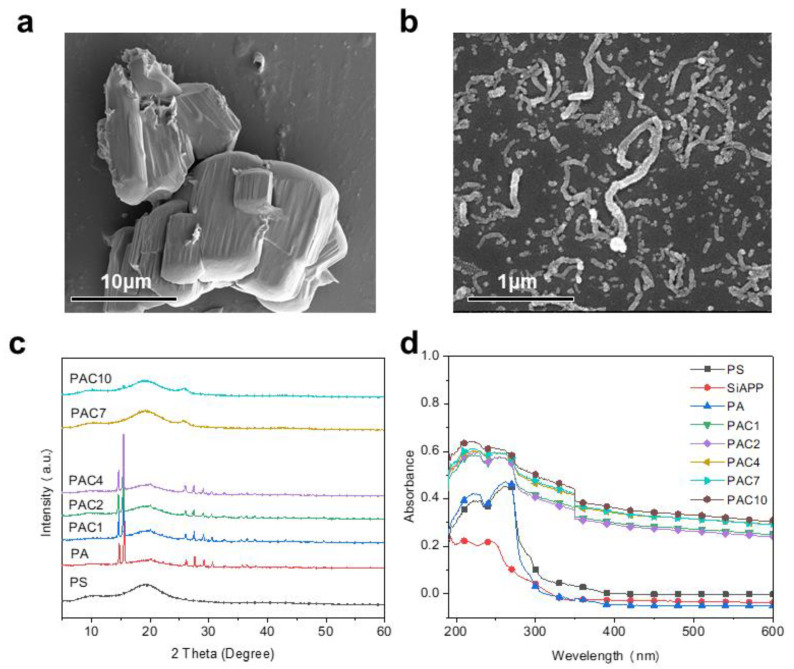
SEM images of (**a**) SiAPP and (**b**) aMWCNT and (**c**) XRD patterns and (**d**) UV-vis absorption spectra of PS and its composites.

SEM was used to examine the morphology of SiAPP and aMWCNT and their dispersion in the PS matrix, as shown in Figure 1. The surface of SiAPP is relatively smooth, and aMWCNT has a tubular structure (see Figure 1a,b). Figure 1c exhibits the XRD patterns of PS composites. It is found that the diffraction peak of PS appears at 19.6°, which is in accordance with the 2θ value of PS in the prior publication [38]. After the addition of SiAPP, the diffraction peaks situated at 14.7°, 15.5°, 26.1° 27.5°, 29.1°, and 30.6° are the characteristic diffraction peaks of SiAPP, which is due to the successful attachment of SiAPP onto the PS spheres [24]. With the incorporation of SiAPP and aMWCNT onto PS, it is found that the characteristic diffraction peaks of PAC1, PAC2, and PAC4 composites are essentially similar to those of PA composites. Significantly, the peaks of PAC7 and PAC10 change, and the peak of SiAPP essentially disappears. In addition, the characteristic diffraction peak located at 26° belongs to aMWCNT, which is due to the low aMWCNT content in PAC1, PAC2, and PAC4 composites, resulting in the diffraction peak of aMWCNT being covered by the diffraction peak of SiAPP. These phenomena indicate that aMWCNT is wrapped on the PS composites. As the loading of aMWCNT increases and the content of SiAPP decreases, the characteristic diffraction peak of aMWCNT appears and the characteristic diffraction peak of SiAPP disappears, demonstrating the successful preparation of PAC composites. The UV-vis absorption spectra were applied to further reveal the variation of SiAPP and aMWCNT on the surface of PS spheres, as presented in Figure 1d. In the range of 200–280 nm, the PAC composites show gradually reduced absorbance with the increasing loading level of aMWCNT, indicating that SiAPP and aMWCNT have good dispersion on PS spheres [15].

**Figure 2:**
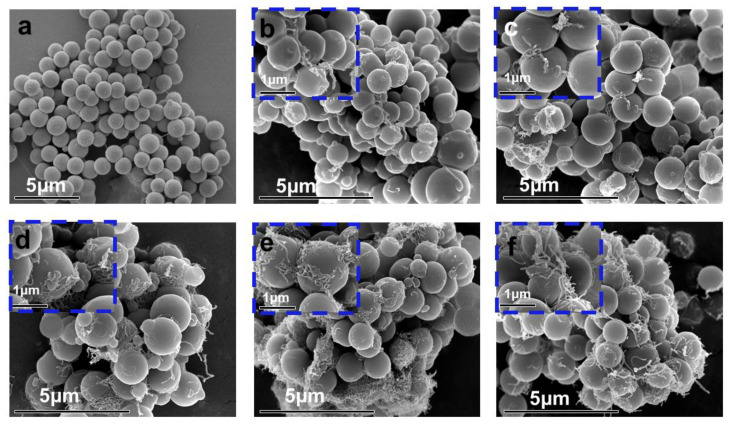
SEM images of (**a**) PS spheres, (**b**) PAC1, (**c**) PAC2, (**d**) PAC4, (**e**) PAC7, and (**f**) PAC10 before hot-pressing.

**Figure 3:**
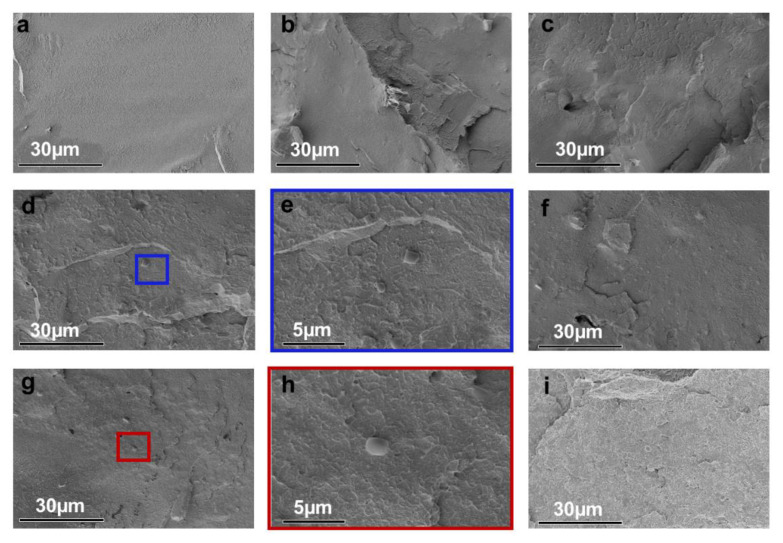
SEM images of fracture surface of (**a**) PS, (**b**) PA, (**c**) PAC1, (**d**,**e**) PAC2, (**f**) PAC4, (**g**,**h**) PAC7, and (**i**) PAC10 after hot-pressing.

For further analysis of the dispersion of SiAPP and aMWCNT on the PS matrix and their interfacial interactions with PS, the SEM images of PS samples before and after hot-pressing were provided, as presented in Figure 2 and Figure 3, respectively. As shown in Figure 2a, the pure PS spheres exhibit fairly smooth surfaces. After the introduction of SiAPP and aMWCNT, the spheres become rough, and their diameters become larger, which is attributed to SiAPP and aMWCNT successfully coating the surface of PS spheres, and it can be seen that there are obvious tube species wrapped on the surface of PS spheres (see Figure 2c–f). With the addition of aMWCNT, the slight agglomeration phenomenon can be observed from the surface of PS spheres. This result is ascribed to the poor dispersion of aMWCNT in the PS matrix and the deterioration of the interfacial interaction between aMWCNT and PS (see Figure 3g). The above results further indicate that SiAPP and aMWCNT are successfully added to the PS matrix.

### 2.2. Thermal Stability Analysis of PS Composites

**Figure 4:**
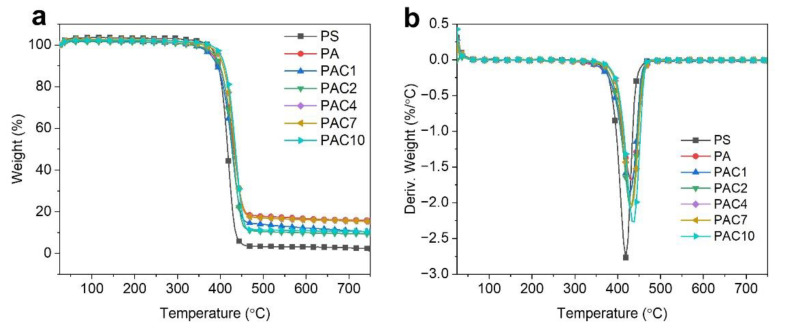
(**a**) TG and (**b**) derivative TG curves of PS and its composites.

The thermal degradation behavior of PS and its composites under nitrogen conditions was carried out using the thermogravimetric analysis (TGA) technique, and the related results are shown in Figure 4 and Table 1. *T*_-10_ and *T*_-max_ are defined as the initial decomposition temperature at 10% weight loss and the temperature at the maximal weight loss rate, respectively. The pure PS displays a one-step decomposition process ranging from 380 to 420 °C due to the thermal decomposition of the polymer chains [39]. It is noted that the PS composites also undergo a one-step decomposition process, indicating that the addition of a flame retardant does not change the thermal degradation behavior of PS. Pure PS starts to degrade at 394.2 °C with basically no residue left. With the incorporation of SiAPP, the values of *T*_-10_ of the PS composites are unchanged. However, values of the *T*_-max_ of the PA composites are increased by 20.0 °C, and the residual mass percentages of the PA composite are 15.8 wt.%, which increased by 565% compared to that of pure PS. After the addition of SiAPP and aMWCNT, the thermal stability of PS is obviously improved. For instance, the *T*_-10_ and *T*_-max_ of PAC10 are improved by 15.0 °C and 25.0 °C, respectively, compared to those of primitive PS. Moreover, the char residues of PAC10 are 10.4 wt.%, representing a 352% increase compared to that of pure PS (2.3 wt.%). The results indicate that dense char residues can prevent heat transfer and delay the thermal degradation of PS and thus improve the thermal stability of PS.

### 2.3. Flame Retardancy Evaluation of PS Composites

**Figure 5:**
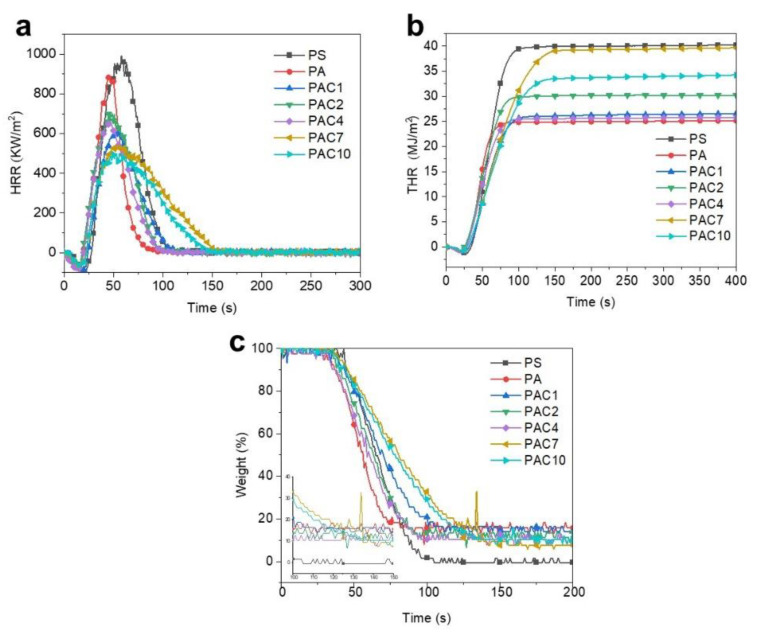
(**a**) HRR, (**b**) THR, and (**c**) weight loss curves of PS and its composites.

**Figure 6:**
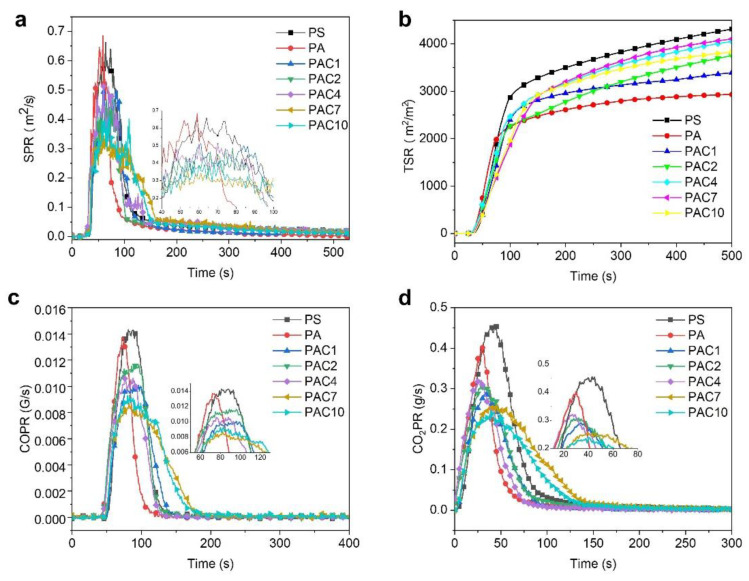
(**a**) SPR, (**b**) TSR, (**c**) COPR, and (**d**) CO2PR curves of PS and its composites.

The cone calorimetry testing (CCT) was widely employed to investigate the combustion performance of polymers with the principle of oxygen consumption, which refers to the production of essentially the same amount of oxygen per unit mass consumed when a substance is completely burned. For combustion tests, the sample was placed on a “cone”-shaped radiant heater and exposed to an external heat flux of 35 kW/m^2^. The important combustion parameters of PS composites obtained from the CCT include the heat release rate (HRR), total heat release (THR), smoke production rate (SPR), total smoke production (TSR), CO production rate (COPR), CO_2_ production rate (CO_2_PR), and weight loss, and the related results are shown in Figure 5 and Figure 6 and Table 2. It can be seen from Figure 5a,b that the pure PS shows a high peak of HRR (PHRR, 994 KW/m^2^) and THR (40 MJ/m^2^). Furthermore, the residual mass of pure PS is almost negligible after combustion (see Figure 5c). After the addition of SiAPP, both PHRR and THR of the PS composites decline to 882 kW/m^2^ and 25 MJ/m^2^, respectively. With the incorporation of SiAPP and aMWNCT, the flame retardancy of the PS composite is significantly improved. For instance, the PHRR of the PAC10 composite decreases to 460 kW/m^2^, which is reduced by 53.7% in comparison with that of pure PS. In addition, the char residues values of PAC10 reach 10.36 wt.%. These results indicate that the improvement in flame retardancy of PS composites is caused by the synergistic effect between SiAPP and aMWNCT.

PS, as a pendant aromatic group-containing polymer, will release abundant smoke and toxic substances during the combustion process, which is a risk to human health and can cause personnel casualties [40,41,42]. Therefore, it is great importance to investigate the release of smoke and toxic substances during the combustion of PS samples. The CO and CO_2_ release profiles and relevant data of toxic fumes for PS and its composite are presented in Figure 6 and Table 2, respectively. As shown in Figure 6a,b, the PSPR and TSR of pure PS are as high as 0.66 m^2^/s and 4354 m^2^/m^2^, respectively. As for PAC composites, the values of PSPR and TSR are obviously reduced. For example, the PSPR and TSR of the PAC10 composite are decreased to 0.36 m^2^/s and 3868 m^2^/m^2^, reduced by 45.5% and 11.2%, respectively, in comparison with those of pure PS. CO and CO_2_ are the two main toxic and suffocating gases that cause casualties in fires. Thus, it is important to discuss the production of CO and CO_2_. The pure PS has a high peak of COPR (PCOPR, 0.0143 g/s) and a peak of CO_2_PR (PCO_2_PR, 0.4552 g/s), which can be observed in Figure 6c,d. Compared to the values of pure PS, both PCOPR and PCO_2_PR of the PA composites declined to 0.0137 kW/m^2^ and 0.4012 MJ/m^2^, respectively. Furthermore, PAC composites show great advantages in the suppression of volatile gas production. Compared with those of pure PS, the PCOPR and PCO_2_PR of the PAC10 composite are greatly decreased to 0.0083 g/s and 0.2190 g/s, respectively. The results above further demonstrate that the synergistic effect between SiAPP and aMWCNT can markedly inhibit the release of smoke and toxic gases.

**Figure 7:**
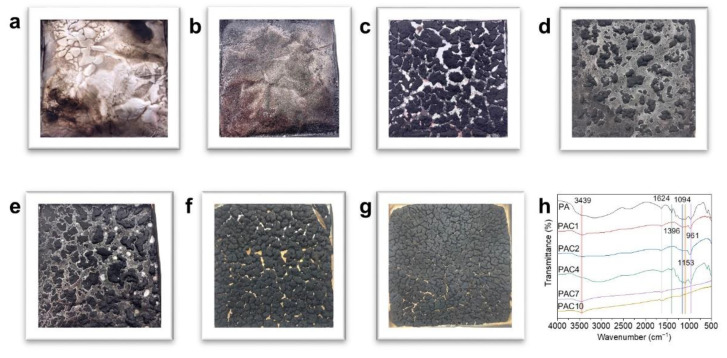
Digital photographs of (**a**) PS, (**b**) PA, (**c**) PAC1, (**d**) PAC2, (**e**) PAC4, (**f**) PAC7, and (**g**) PAC10 after CCT and (**h**) FTIR spectra of the char residues of PS composites.

The formation of compact and continuous char residues can effectively improve the flame retardancy of polymers [18,42]. Therefore, it is necessary to further research the char residues of PS composites after combustion. The digital photographs of PS composites are presented in Figure 7a–g. It can be clearly seen that pure PS has almost no char residues left after combustion, while the PA composite shows a few char residues. When 1 wt.% aMWCNT is added, the PAC1 composite has clearly discontinuous and loose char residues. When the content of aMWCNT further increases, the amount of char residues increases significantly and these char residues become more compact and denser. The production of stable char residues is mainly a result of the aMWCNT catalyzing the PS matrix into char [17,42,43]. This result is consistent with the result obtained from Figure 4.

In order to further analyze the chemical structure of the char residuals of PS composites, the char residuals of PS composites were tested by FTIR, as presented in Figure 7h. The characteristic absorption peak situated at 3439 cm^−1^ belongs to the stretching vibration of -OH of carboxyl in aMWCNT [44]. The absorption peaks at 1624 cm^−1^ and 1390 cm^−1^ correspond to the stretching vibration of C=C and the stretching vibration of C=O, respectively [45]. The peak attributed to the stretching vibration of Si-O-Si occurs at 1153 cm^−1^ [23]. The absorption peaks identified as the stretching vibrations of Si-O-C are located at 1094 cm^−1^ and 961 cm^−1^ [22]. These results indicate that the PAC composites catalyze the formation of a large number of char residuals during combustion, which can act as a barrier to prevent the diffusion of combustible gas and protect the PS base from high-temperature thermal radiation.

**Figure 8:**
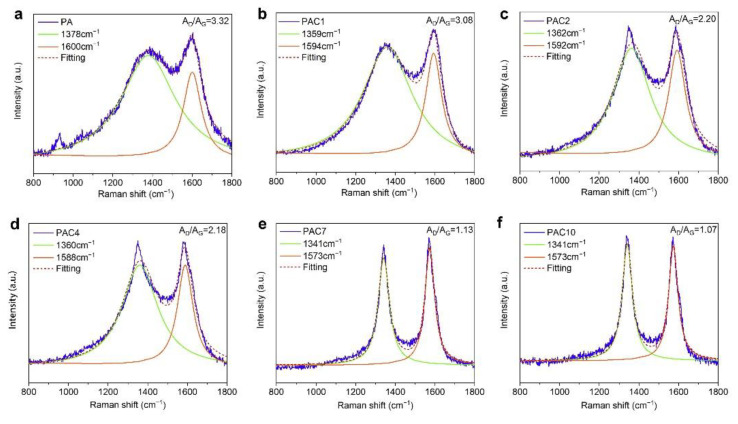
Raman spectra of the char residues of PS composites after CCT: (**a**) PA, (**b**) PAC1, (**c**) PAC2, (**d**) PAC4, (**e**) PAC7, and (**f**) PAC10.

In order to obtain more information about the char residues of PS composites, the chemical structure of char residues was further analyzed by the Raman technique. As plotted in Figure 8, the Raman spectra indicate that the characteristic peaks of all the samples appear at approximately 1360 cm^−1^ and 1580 cm^−1^. The former band (the D band) is the disordered vibration peak of graphene, and the latter (the G band) is considered as in-plane vibration of the sp^2^ atom [46,47]. Generally, the graphitization degree of char residues is evaluated according to the integral area ratio (*A*_D_/*A*_G_) of the D band to the G band. The lower the value of *A*_D_/*A*_G_, the higher the degree of graphitization of char residues is, which is more conducive to suppressing the generation of heat, smoke, and toxicity of polymers. The values of *A*_D_/*A*_G_ are arranged as follows: PAC10 (1.07) < PAC (1.13) < PAC4 (2.18) < PAC2 (2.20) < PAC1 (3.08) < PA (3.32), indicating that PA has the largest *A*_D_/*A*_G_ value, while the *A*_D_/*A*_G_ value of PAC is the smallest. The result means that the char residues of PAC10 display the best flame-retardant effect. This is consistent with the results of the FTIR analysis.

**Figure 9:**
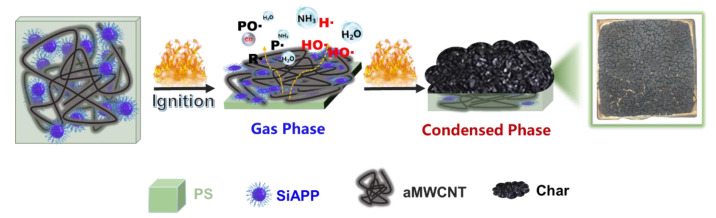
Schematic illustration for the proposed flame-retardant mechanism.

Based on the above analysis, the flame-retardant mechanism of PAC composites is shown in Figure 9. The improvement in flame-retardant properties of PAC composites is attributed to the combination of the condensed phase and the gas phase. The radical capture effect of SiAPP is crucial to improving the thermal stability, flame retardancy, and smoke suppression of PS. SiAPP decomposes to generate phosphate and free radicals containing phosphate (PO_2_• and PO•) [48]. These free radicals are able to enter the gas phase and capture radicals such as H• and HO•, interrupting the combustion reaction [49,50,51]. Moreover, the introduction of aMWCNT further enhances the flame-retardant properties. The synergistic effect between SiAPP and aMWCNT can catalyze the generation of robust char layers [45], which can block the heat transfer and combustible gas migration, and retard the thermal decomposition of PS composites. When the PS composites are burned, SiAPP and aMWCNT will produce magnanimous inert gases (NH_3_, H_2_O, and CO_2_, etc.), which decrease the concentration of oxygen and combustible substances, and terminates the combustion. Therefore, the flame-retardant properties of PS can be improved.

### 2.4. EMI Shielding Performance Estimation of PS Composites

**Figure 10:**
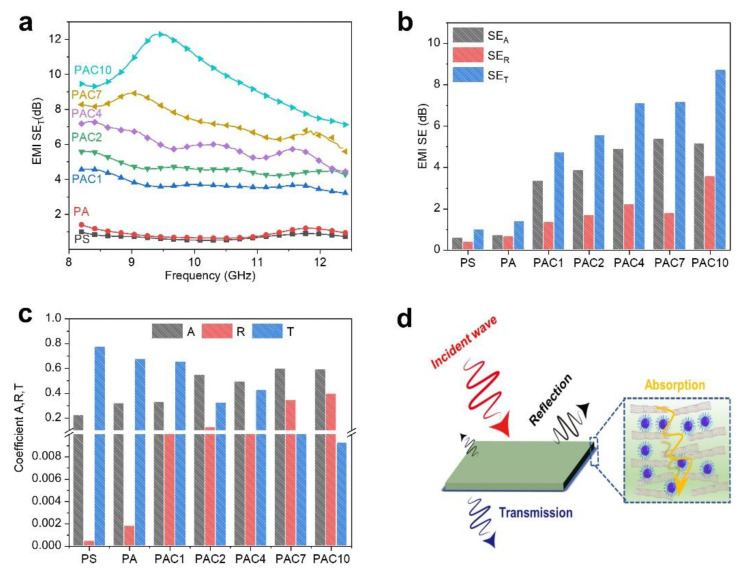
(**a**) EMI SE curves (**b**) the SEA, SER, and SET values; (**c**) the A coefficient, R coefficient, and T coefficient of the PS composites in the X band (8.2–12.4 GHz); (**d**) EMI shielding mechanism of the PS composites.

The EMI SE curves of PS and its composites are shown in Figure 10a. It is obvious that PS and PA have low EMI SE because PS as an insulator does not block any electromagnetic waves, resulting in continued propagation of the electromagnetic waves directly across the material. After adding 1 wt.% aMWCNT, the electromagnetic shielding properties of the PS composites are all improved. For instance, the EMI SE of PAC1 is approximately 4 dB. Moreover, the electromagnetic shielding performance is gradually improved with the increase in aMWCNT. With the introduction of 10 wt.% aMWCNT, the EMI SE of PAC10 can reach 12 dB. In addition, it can be clearly observed from Figure 10b that PAC composites show higher SE_A_ than SE_R_. The above conclusions imply that the attenuation of electromagnetic waves is mainly dominated by the absorption of external electromagnetic waves and is assisted by multiple reflections. In order to further study the mechanism of EMI shielding of PS composites, the absorption coefficient (A), reflection coefficient (R), and transmission coefficient (T) of PS composites are shown in Figure 10c. It is found that the A coefficient is higher than the R coefficient, which is consistent with the SE_A_, SE_R_, and SE_T_ values. These results further prove that absorption is the main mechanism of electromagnetic wave dissipation. Therefore, the mechanism of electromagnetic shielding of PAC composites is illustrated in Figure 10d. The aMWCNT, as a typical one-dimensional conductive material, is able to form an interconnected conductive network after being added to the PS matrix, which can intercept electromagnetic radiation [52,53]. The majority of the electromagnetic waves enter the interior of the PS composites, and a small part of them are reflected due to the increasing impedance mismatches between the air and PS composites’ surface [54,55]. The conductive network formed by the addition of aMWCNT to the PS matrix provides more pathways for the transmission of electromagnetic waves, which causes the electromagnetic waves that enter the interior of the composite to be absorbed and dissipated [56]. Increasing the frequency of multiple reflections and the reabsorption of electromagnetic waves promote the capacity of PS composites to absorb electromagnetic waves [57]. Therefore, the one-dimensional conductive material (i.e., aMWCNT) improves the electromagnetic shielding performance of PS composites.

## 3. Conclusions

In this work, PS/SiAPP/aMWCNT composites were successfully prepared using methods of suction filtration and hot-pressing. The results of morphology and structure characterization indicated that SiAPP and aMWCNT had good dispersion in PS and showed excellent compatibility with the PS matrix. After the introduction of SiAPP and aMWCNT, the thermal stability of PS was significantly improved. For instance, the *T*_-10_ and *T*_-max_ of the PAC10 were improved by 15.0 °C and 25.0 °C, respectively, compared to those of pure PS. The CCT results showed that the PHRR, PCOPR, and PSPR of PAC10 decreased by 53.7%, 41.9%, and 45.5%, respectively, compared with those of native PS. The improvement in flame-retardant and smoke inhibition properties of PS is principally attributed to SiAPP thermally decomposed to produce free radicals (PO_2_• and PO•), which can react with H• and HO• to produce stable compounds. Furthermore, the fire hazard of PS was significantly reduced due to the aMWCNT synergized with SiAPP to promote the generation of compact char residues. Moreover, the addition of aMWCNT significantly improved the electromagnetic shielding properties of the PS composites. The EMI SE of PAC10 could reach 12 dB, and the attenuation of the electromagnetic wave was dominated by the absorption. This work provides an alternative methodology to fabricating PS materials with higher fire safety and electromagnetic shielding properties.

## 4. Materials and Methods

### 4.1. Raw Materials

Styrene (≥99.5%, AR), potassium peroxydisulfate (≥99.5%, AR), anhydrous ethanol (≥99.7%, AR), and sulfuric acid (95.0–98.0%, AR) were purchased from Sinopharm Chemical Reagent Co., Ltd. (Shanghai, Shanghai, China). Sodium dodecylbenzene sulfonate (SDBS) was supplied by Tianjin Fuchen Chemical Reagents (Tianjin, Tianjin, China). Nitric acid (65.0–68.0%, AR) was offered by Xilong Scientific Co., Ltd. (Shantou, Guangdong, China). SiAPP (203) was provided by Sichuan Fine Chemical Co., Ltd. (Chengdu, Sichuan, China). MWCNTs (a diameter of 20–40 nm, a length of >5 mm, and a special surface area of 40–70 m^2^/g) were obtained from Shenzhen Nanotech Port Co., Ltd. (Shenzhen, Guangdong, China). Nitrogen (≥99.999%) was purchased from Fuzhou Xinhang Industrial Gas Co., Ltd. (Fuzhou, Fujian, China). Both styrene and potassium peroxydisulfate were further purified prior to use. The high-speed centrifuge was TG16G, which was received from Shanghai Anting Scientific Instrument Factory (Shanghai, Shanghai, China). The type of ultrasonic cleaner was KQ-600KDV, which was afforded by Kunshan Ultrasonic Instrument Co., Ltd. (Kunshan, Jiangsu, China). The type of vulcanizing machine was CREE-6061, which was bought from Kerry Instrument Technology Co., Ltd. (Dongguan, Guangdong, China).

### 4.2. Synthesis of the PS Spheres

The PS spheres were synthesized according to previous literature [55,57,58]. Typically, 0.44 g of SDBS and 200 mL of anhydrous ethanol were poured into a 500 mL three-necked flask with mechanical stirring to form solution A. Then, 0.4 g of potassium persulfate was dissolved in 80 mL of deionized water via rapid agitation to obtain solution B. Afterwards, solution B was slowly added to solution A under a nitrogen atmosphere at room temperature. Then, 18 mL of styrene was dropwise added into the abovementioned solution. Then the mixture was maintained at 80 °C for 8 h. Then, 2 mL of styrene was added dropwise into the mixture and was stirred for 12 h. It was required to add high-purity nitrogen to prevent the styrene from being oxidized during the whole experiment. Finally, the resulting product was centrifuged at 8000 rpm for 5 min, washed several times with ethanol, and dried at 70 °C overnight.

### 4.3. Acidification of MWCNT

The modification of MWCNT was carried out via acidification. Firstly, 2 g of untreated MWCNT was added to a 400 mL mixed solution containing 300 mL of H_2_SO_4_ and 100 mL of HNO_3_ under ultrasonic agitation for 2 h. The ultrasound frequency was 40 KHZ during the reaction. Then, the mixed solution was stirred mechanically at 80 °C for 4 h. After being cooled to room temperature, the well-dispersed solution was washed with deionized water until its pH value was ca. 7.0, and was labelled aMWCNT.

### 4.4. Fabrication of PS/SiAPP/aMWCNT Composites

**Figure 11:**
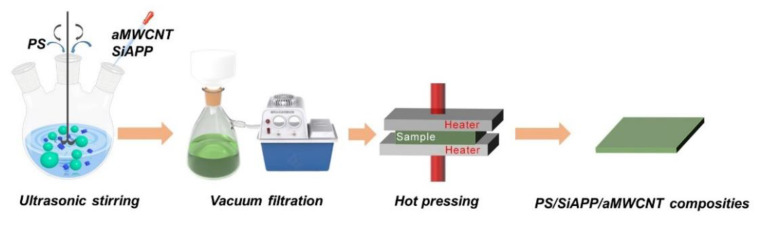
The schematic process for fabrication of PS/SiAPP/aMWCNT composites.

The PS/SiAPP/aMWCNT composites were prepared via vacuum-assisted filtration and hot-pressing methods. The preparation process of PS composites is illustrated in Figure 11. Firstly, the PS spheres and SiAPP powder were poured into 200 mL of deionized water and subsequently ultrasound-assisted agitated for 1 h to obtain a dispersion. Then 0.40 g of aMWCNT was dropwise added into the dispersion under ultrasound-assisted agitation for 1 h. After filtering and drying, the PS composite was obtained by hot-pressing at 190 °C under 10 MPa. The PS sample with aMWCNT content of 1 wt.% was marked as PAC1 (among them P, A, and C stand for PS, SiAPP, and aMWCNT, respectively). The final acquisition of various content samples involved PS, PA, PAC1, PAC2, PAC4, PAC7, and PAC10. Detailed information on PS/SiAPP/aMWCNT composites is shown in Table 3.

### 4.5. Characterization

The morphologies and microstructures of the additives and fracture surfaces of PS samples were observed via scanning electron microscopy (SEM, FEI Nova NanoSEM 230, FEI CZECHREPUBLIC S.R.O., Brno, Czech Republic). The sample was sputter-coated with a thin gold layer. X-ray diffraction (XRD) patterns were acquired via a DY1602/Empyrean X-ray diffractometer (Panalytical, Almelo, Netherlands) with Cu Kα radiation (λ = 1.54178 Å). Additives dispersed in ethyl alcohol were dropped onto a Cu grid. The Fourier transform infrared (FTIR) spectrum was tested using a Nicolet 50 spectrometer (Nicolet Instrument, Madison, WI, USA) with a range of 400–4000 cm^−1^. UV-vis absorption spectra were provided using a UV-vis spectrophotometer (SOLID 3700, Shimadzu, Japan) at room temperature. Raman spectra were observed by a Renishaw Invia Raman Microscope (Invia Reflex, Renishaw In via, Wotton-under-Edge, UK) with a wide scope of 200 to 2000 cm^−1^. TGA was measured via a thermal analyzer (TA Q5000, TA Co., New Castle, DE, USA) from 50 to 750 °C with a heating rate of 20 °C/min under a nitrogen atmosphere. The CCT was conducted on a TTech-GBT16172-2 type cone calorimeter (TESTech, Suzhou, China) under an external heat flux of 35 kW/m^2^. The dimensions of each sample were 100 × 100 × 1 mm^3^, and it was wrapped in a layer of aluminum foil before testing. The EMI SE of PS composites in the frequency range of 8.2–12.4 GHz (X-band) was determined with an N5222B vector network analyzer (Keysight Technologies, Santa Rosa, CA, USA) at room temperature. The specimen was cut into a rectangle with dimensions of 25.6 × 11.2 mm^2^.

## Figures and Tables

**Table 1:** The related thermal data of PS and its composites under nitrogen conditions.

Sample No.	*T*_-10_ (°C)	*T*_-max_ (°C)	Residue at 750 °C (wt.%)
PS	394.2	448.1	2.3
PA	394.2	468.2	15.3
PAC1	394.2	463.3	10.2
PAC2	399.1	468.5	9.3
PAC4	404.1	468.9	15.8
PAC7	404.3	468.3	15.3
PAC10	409.2	473.2	10.4

**Table 2:** The CCT data of PS and its composites at a heat flux of 35 kW/m^2^.

Sample No.	PHRR (KW/m^2^)	THR (MJ/m^2^)	PCOPR (g/s)	COTY (kg/kg)	PCO_2_PR (g/s)	PSPR (m^2^/S)	TSR (m^2^/m^2^)	Weight Loss (wt.%)
PS	994	40.0	0.0143	0.6607	0.4552	0.66	4354	0.24
PA	882	25.0	0.0137	0.4517	0.4012	0.67	2919	15.18
PAC1	603	26.1	0.0098	0.5674	0.2850	0.46	3446	16.18
PAC2	700	29.9	0.0109	0.6491	0.0301	0.47	3798	13.51
PAC4	647	25.6	0.0098	0.5330	0.3172	0.51	4050	10.87
PAC7	530	39.2	0.0082	0.6915	0.2430	0.34	4106	9.19
PAC10	460	33.7	0.0083	0.5694	0.2190	0.36	3868	10.36

Notes: The PHRR, THR, PCOPR, COTY, PCO_2_PR, PSPR, and TSR refer to peak heat release rate, total heat release, peak carbon monoxide production rate, total carbon monoxide yield, peak carbon dioxide production rate, total carbon dioxide yield, peak smoke production rate, and total smoke release, respectively.

**Table 3:** Formulation of PS/SiAPP/aMWCNT composites.

Sample No.	PS (g)	SiAPP (g)	aMWCNT (g)
PS	40.00	0.00	0.00
PA	32.00	8.00	0.00
PAC1	32.00	7.60	0.40
PAC2	32.00	7.20	0.80
PAC4	32.00	6.40	1.60
PAC7	32.00	5.20	2.80
PAC10	32.00	4.00	4.00

## Data Availability

The data are not publicly available due to the confidentiality requirement for following research work.
